# Immunogenicity of ChAdOx1 nCoV-19 vaccine after a two-dose inactivated SARS-CoV-2 vaccination of dialysis patients and kidney transplant recipients

**DOI:** 10.1038/s41598-022-07574-w

**Published:** 2022-03-04

**Authors:** Jackrapong Bruminhent, Chavachol Setthaudom, Rungthiwa Kitpermkiat, Sasisopin Kiertiburanakul, Kumthorn Malathum, Montira Assanatham, Arkom Nongnuch, Angsana Phuphuakrat, Pongsathon Chaumdee, Chitimaporn Janphram, Sansanee Thotsiri, Piyatida Chuengsaman, Sarinya Boongird

**Affiliations:** 1 Division of Infectious Diseases, Department of Medicine, Faculty of Medicine Ramathibodi Hospital, Mahidol University, Bangkok, Thailand; 2 Ramathibodi Excellence Center for Organ Transplantation, Faculty of Medicine Ramathibodi Hospital, Mahidol University, 270 Rama VI Road, Ratchathewi, Bangkok, 10400 Thailand; 3 Immunology Laboratory, Department of Pathology, Faculty of Medicine Ramathibodi Hospital, Mahidol University, Bangkok, Thailand; 4 Division of Nephrology, Department of Medicine, Faculty of Medicine Ramathibodi Hospital, Mahidol University, Bangkok, 10400 Thailand; 5 Department of Medicine, Faculty of Medicine Ramathibodi Hospital, Mahidol University, Bangkok, Thailand; 6 Somdech Phra Debaratana Medical Center, Faculty of Medicine Ramathibodi Hospital, Mahidol University, Bangkok, Thailand; 7 Banphaeo Dialysis Group, Banphaeo-Charoenkrung Peritoneal Dialysis Center, Banphaeo Hospital, Bangkok, Thailand

**Keywords:** Infectious diseases, Nephrology, Infectious diseases

## Abstract

Vaccination with inactivated SARS-CoV-2 virus produces suboptimal immune responses among kidney transplant (KT), peritoneal dialyzed (PD), and hemodialyzed (HD) patients. Participants were vaccinated with two-dose inactivated SARS-CoV-2 vaccine (V2) and a third dose of ChAdOx1 nCoV-19 vaccine (V3) at 1–2 months after V2. We enrolled 106 participants: 31 KT, 28 PD, and 31 HD patients and 16 controls. Among KT, PD, and HD groups, median (IQR) of anti-receptor binding domain antibody levels were 1.0 (0.4–26.8), 1092.5 (606.9–1927.2), and 1740.9 (1106–3762.3) BAU/mL, and percent neutralization was 0.9 (0–9.9), 98.8 (95.9–99.5), and 99.4 (98.8–99.7), respectively, at two weeks after V3. Both parameters were significantly increased from V2 across all groups (*p* < 0.05). Seroconversion and neutralization positivity rates in PD, HD, and control groups were 100% but were impaired in KT patients (39% and 16%, respectively). S1-specific T-cell counts were increased in PD and HD groups (*p* < 0.05) but not in KT patients. The positive S1-specific T-cell responder rate was > 90% in PD, HD, and control groups, which was higher than that in KT recipients (74%, *p* < 0.05). The heterologous inactivated virus/ChAdOx1 nCoV-19 vaccination strategy elicited greater immunogenicity among dialysis patients; however, inadequate responses remained among KT recipients (TCTR20210226002).

## Introduction

Coronavirus disease 2019 (COVID-19) is an infectious disease that has caused a worldwide pandemic. Clinical presentation ranges from asymptomatic or mild upper respiratory tract disease to pneumonia and respiratory failure^1^. Patients who have comorbidities or are taking immunosuppressive drugs are likely to develop severe disease and complications^2^. Patients with end-stage kidney disease (ESKD) requiring dialysis often have compromised humoral immunity (HMI) because of uremic toxins, inflammation, and malnutrition. Furthermore, kidney transplant (KT) recipients have impaired cell-mediated immunity (CMI) from taking immunosuppressants to maintain their allografts^3,4^. Therefore, a preventive strategy is recommended to prepare for potentially aggressive disease. Immunogenicity of vaccine platforms and individual immune status are variable^5^. When whole, inactivated SARS-CoV-2 virus was utilized as a vaccine nationwide, optimal immune responses were observed among immunocompetent individuals^6^. However, the CVIM1 and ICON1 studies were prospective studies that investigated SARS-CoV-2-specific HMI and CMI responses after vaccination with two doses of inactivated SARS-CoV-2 vaccine in KT and ESKD patients who underwent hemodialysis (HD) and peritoneal dialysis (PD), respectively. SARS-CoV-2-specific HMI responses were suboptimal in dialysis patients and substantially impaired in KT patients compared with the CMI response^7,8^.

The WHO released interim recommendations for a third COVID-19 vaccine dose for immunocompromised individuals^9^. Investigations of third doses have primarily used homologous vaccine platforms and evaluated mRNA- and adenoviral vector-based vaccines. A heterologous vaccine platform with two doses of inactivated SARS-CoV-2 vaccine and a third dose of ChAdOx1 nCoV-19 vaccine was proposed to enhance immune responsiveness among immunocompromised populations such as patients receiving active treatment for hematologic malignancies or patients with advanced HIV infection. However, the immunogenicity and safety studies among KT and dialysis patients vaccinated with the third dose of ChAdOx1 nCoV-19 vaccine have recently received attention. Thus, we evaluated the immunogenicity and safety profiles of this new vaccination regimen among these vulnerable individuals.

## Materials and methods

### Study design

A single-arm clinical trial (CVIM2/ICON2 study) was performed between July 2021 and September 2021 at Ramathibodi Hospital, Mahidol University, Bangkok, Thailand. Participants who were vaccinated with two doses (V2) of the inactivated SARS-CoV-2 (CoronaVac, 3 μg of inactivated whole-virus SARS-CoV-2 in 0.5 ml) vaccine (Sinovac Biotech Ltd., China), were offered a third dose (V3) of the ChAdOx1 nCoV-19 (AZD1222, ChAdOx1-S of at least 2.5 × 10^8^ infectious units in 0.5 ml) vaccine (Oxford University-AstraZeneca) at 1 to 2 months after V2 (Supplementary Fig. 1). We assessed both HMI and CMI responses and monitored for adverse events (AEs) following the third dose of the ChAdOx1 nCoV-19 vaccine.

The Institutional Review Board of the Faculty of Medicine, Ramathibodi Hospital, Mahidol University, Bangkok, Thailand, approved the study protocol (approval number: MURA2021/523). The study was conducted following the principles laid out in the Declaration of Helsinki and was registered with the Thai Clinical Trials Registry (TCTR202110524002). All participants provided written informed consent. The approval from the Faculty of Medicine Ramathibodi Hospital was applied with acknowledgment of the medical director of Banphaeo General Hospital (Charoenkrung branch) due to unavailability of the local ethic committee of this facility. Furthermore, the patients from Banphaeo General Hospital (Charoenkrung branch) agreed to the participation, the referral, and providing information about their medical condition.

The eligibility criterion for participation (Supplementary Fig. 5) was adults aged 18 to 59 years old. Those with suspected respiratory tract infections within the past three days, ongoing infections, and previous history of COVID-19 were excluded. Screening questions were used to assess concurrent respiratory tract infection and potential exposure to COVID-19. Nasopharyngeal and oropharyngeal swabs were not collected for SARS-CoV-2 reverse transcription polymerase chain reaction (RT-PCR) tests before vaccination. Dialysis patients were those who were stable on their dialysis prescriptions for at least one month. KT recipients were at least 1-month post-transplant with stable allograft function and immunosuppressive regimens and without recent allograft rejections or intensive immunosuppressant therapy. Intense immunosuppressant regimens included methylprednisolone pulse therapy (500 mg/day for three days), antithymocyte globulin therapy, rituximab therapy within six months, or ≥ 15 mg/day prednisolone. A low C_0_ trough level of calcineurin inhibitor (CNI) was defined as ≤ 5 ng/mL tacrolimus or ≤ 150 ng/mL cyclosporine^10^. A low dose of mycophenolic acid (MPA) was defined as ≤ 1 g/day mycophenolate mofetil (MMF) or ≤ 720 mg/day mycophenolate sodium (MPS)^11^.

HMI and CMI responses were assessed two weeks after V2 and V3. HMI was measured using a SARS-CoV-2 immunoglobulin G (IgG) assay, which tests for antibodies against the S1 receptor-binding domain (RBD) of the SARS-CoV-2 spike protein, and SARS-CoV-2 surrogate virus neutralization test (sVNT). CMI was measured using an enzyme-linked immunospot assay for interferon-γ (IFN-γ).

### SARS-CoV-2 anti-RBD IgG assay

SARS-CoV-2 anti-RBD IgG antibodies were measured using the Abbott SARS-CoV-2 IgG II Quantification assay (Abbott Diagnostics, USA). Plasma samples were run on the Abbott Alinity instrument following the manufacturer’s instructions. The assay is a chemiluminescent microparticle immunoassay for the quantitative detection of IgG against the RBD of the SARS-CoV-2 spike protein in human serum. The sequence of the RBD was derived from the WH-Human 1 coronavirus. The quantitative results for anti-RBD IgG were reported in binding antibody units (BAU)/mL. A cut-off value of ≥ 7.1 BAU/mL was considered seroconversion^12^. This value provided a diagnostic sensitivity and specificity of 91.6% and 99.4%, respectively^12,13^.

### SARS-CoV-2 sVNT

The function of anti-SARS-CoV-2 S1/RBD antibodies was determined using a SARS-CoV-2 NeutraLISA surrogate neutralization assay (Euroimmun, Germany). The neutralizing antibodies in plasma inhibit binding between the RBD and angiotensin-converting enzyme 2 receptor. The sequence of the RBD was also derived from the WH-Human 1 coronavirus. The neutralizing antibody was measured and reported as a percentage; those participants with > 35% inhibition were considered positive^14^. This value provided a sensitivity and specificity of 95.9% and 99.7%, respectively^15^.

### SARS-CoV-2 ELISpot assay

SARS-CoV-2–specific CMI responses were measured using the SARS-CoV-2 ELISpot assay as previously described^7^. The SARS-CoV-2 S1 scanning peptide pool (Mabtech, Inc.) and the SARS-CoV-2 spike protein, nucleoprotein, membrane protein, open reading frame (ORF)-3a, and ORF-7a proteins (SNMO) peptide pool (Mabtech, Inc.) were derived from the WH-Human 1 coronavirus and used as stimulants. Anti-CD3 antibody was a positive control. Results were reported as IFN-γ-producing spot forming units (SFUs) per 10^6^ peripheral blood mononuclear cells (PBMCs) for each peptide pool^16^. Participants with ≥ 6 SFUs/10^6^ PBMCs were considered responders^17,18^.

### Adverse events

All participants were monitored for solicited AEs within 30 min after vaccination (Supplementary Fig. 2) and on days 3 and 7 by telephone (Supplementary Fig. 3). In addition, unsolicited AEs were documented by participants in the provided diaries (Supplementary Fig. 4). The occurrence of adverse events following vaccination was collected and compared.

### Statistical analyses

Categorical and continuous variables were reported as absolute numbers, frequencies, or medians with interquartile range (IQR). The chi-square test and Fisher’s exact test were performed to compare categorical variables as appropriate. The Mann–Whitney U test and Wilcoxon signed-rank test were performed to compare continuous variables between and within the groups, respectively. Analysis of variance was used to compare variables across all groups. Statistical analyses were performed with Stata statistical software, version 15 (StataCorp, LLC; College Station, TX, USA). *P* values < 0.05 were considered significant. The prevalence of anti-RBD IgG, percent neutralization, and S1- or SNMO-specific T-cell SFUs/10^6^ PBMCs were presented as dot plots or bar graphs generated with GraphPad Prism 6.0 (GraphPad Software, Inc.; San Diego, CA, USA).

## Results

### Clinical characteristics of participants

We enrolled 106 participants (Supplementary Fig. 6): 31 KT, 28 PD, and 31 HD patients and 16 healthy controls. Clinical characteristics of all participants are presented in Table 1. Age; sex; Charlson comorbidity index; comorbidities; absolute lymphocyte counts; and blood levels of albumin, calcium, and phosphorus were variable across all groups (*p* < 0.05). KT recipients were slightly older and received immunosuppressants. Two KT recipients (6.5%) received the ChAdOx1 nCoV-19 vaccine within the first year after transplant.

**Table 1:** Clinical characteristics of the study participants.

Characteristics N (%) or median (IQR)	KT (N = 31)	PD (N = 28)	HD (N = 31)	Controls (n = 16)	*p* value
Age, years	51 (42–54)	41 (32–52)	44 (36–54)	41 (38–45)	< 0.01
Male sex	18 (58)	17 (61)	23 (74)	5 (31)	0.05
Body mass index, kg/m^2^	24.1 (22.3–28.3)	22.9 (19.8–26.2)	25.5 (23.3–28.0)	25.6 (22.3–31.1)	0.13
Age-adjusted Charlson Comorbidity Index	3 (2–4)	2 (2–4)	3 (2–5)	0 (0–0)	< 0.01
Comorbidities					
Diabetes mellitus	12 (39)	7 (24)	14 (45)	1 (6)	0.03
Hypertension	22 (71)	25 (86)	24 (77)	2 (13)	< 0.01
Dyslipidemia	3 (10)	10 (34)	11 (35)	4 (25)	0.06
Coronary artery disease	1 (4)	2 (7)	7 (23)	0 (0)	0.04
Etiologies of ESKD					0.02
Diabetic nephropathy	3 (10)	5 (18)	6 (19)	N/A	
Hypertensive nephropathy	1 (3)	8 (28)	3 (10)	N/A	
Chronic glomerulonephritis	6 (19)	8 (28)	5 (16)	N/A	
Other	4 (13)	1 (3)	3 (10)	N/A	
Unknown	17 (55)	7 (24)	14 (45)		
Immunosuppressive drugs	31 (100)	1 (3)	0 (0)	0 (0)	
White blood cell count, × 10^9^/L	7.78 (6.21–9.20)^a^	7.05 (5.66–8.05)	6.91 (5.77–8.08)	6.88 (6.60–8.89)^b^	0.63
Absolute lymphocyte count, × 10^9^/L	1.89 (1.50–2.40)^a^	1.27 (0.94–2.20)	1.59 (1.24–1.95)	1.90 (1.60–2.20)^b^	0.02
Hemoglobin, g/dL	12.5 (11.3–13.6)^a^	10.0 (8.4–11.8)	10.9 (9.6–12.7)	12.9 (12.50–13.70)^b^	< 0.01
Albumin, g/L	41 (40–44)^c^	33 (30–36)	39 (37–42)	N/A	< 0.01
Calcium, mg/dL	9.3 (8.9–9.8)^d^	9.0 (8.0–9.0)	9.0 (8.0–9.6)	N/A	0.02
Phosphorus, mg/dL	3.3 (2.5–3.7)^d^	5.0 (4.0–6.9)	5.0 (3.8–7.0)	N/A	0.02

*ESKD* end-stage kidney disease, *HD* hemodialyzed patients, *IQR* interquartile range, *KT* kidney transplant recipients, *N* number, *N/A* not applicable, *PD* peritoneal dialyzed patients.

^a^Evaluated in 27 participants.

^b^Evaluated in 9 participants.

^c^Evaluated in 25 participants.

^d^Evaluated in 23 participants.

### SARS-CoV-2 specific HMI responses

**Figure 1:**
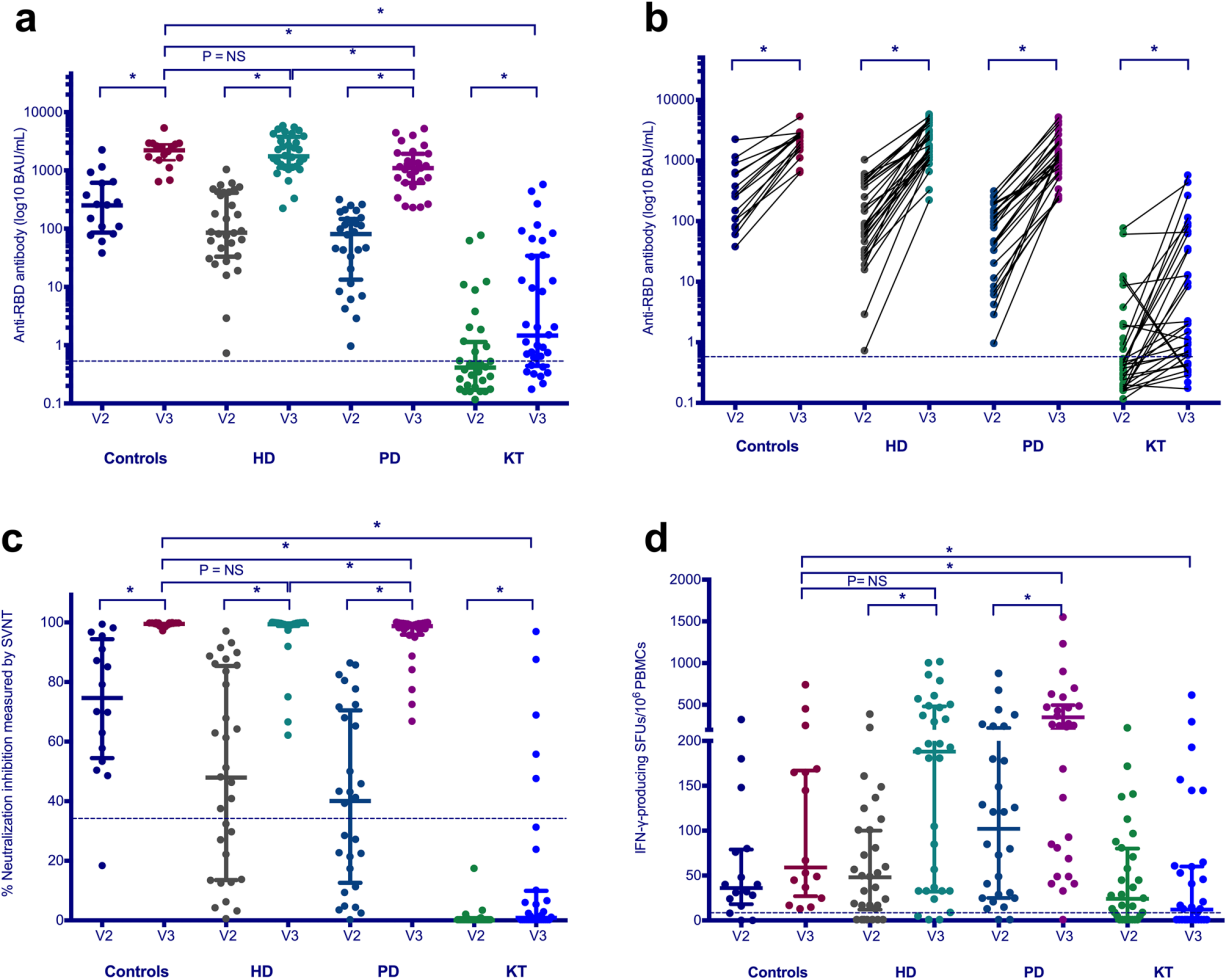
Humoral and cell-mediated immune responses in kidney transplant (KT), peritoneal dialyzed (PD), and hemodialyzed (HD) patients and healthy individuals (controls) who received two doses of inactivated vaccine (V2) compared with those receiving a third dose of ChAdOx1 nCoV-19 vaccine (V3). (**a**) Anti-receptor-binding domain (RBD) antibody levels at two weeks after V2 and V3 are presented in log10 binding antibody units (BAU)/mL using scatter dot plots. Each dot represents an individual participant, and horizontal lines indicate the median and interquartile range. The dotted line indicates the threshold value of 7.1 BAU/mL. (n = 106) (**b**) Anti-RBD antibody levels at two weeks after V2 and V3 within individuals are shown. (n = 106) (**c**) The percent neutralization inhibition at two weeks after V2 and V3 are presented. The dotted line indicates the 35% threshold for neutralization positivity. (n = 106) (**d**) SARS-CoV-2–specific, IFN-γ-producing T-cell responses to the S1 protein at two weeks after V2 and V3 are presented in the scatter dot plots. (n = 106) Horizontal lines indicate the median and interquartile range. The dotted line indicates the threshold value of 6 SFUs/10^6^ PBMCs. *P-value < 0.05. IFN-γ, interferon-γ; SFU, spot forming unit; NS, non-significant; PBMCs, peripheral blood mononuclear cells; S, spike glycoprotein; S1, S1 domain of spike protein.

At two weeks after V3, the median anti-RBD antibody levels among KT, PD, HD, and controls were greater after V3 in all groups compared with those after V2 (*p* < 0.05) **(**Table 2, Fig. 1a,b). Anti- RBD antibody levels after V3 in HD patients were comparable with controls but significantly impaired in PD (*p* < 0.05) and KT (*p* < 0.05) patients. The seroconversion rates were 39% for KT patients and 100% for PD, HD, and control groups. For ESKD patients receiving dialysis, the seroconversion rate improved from 88% after V2 to 100% after V3 (*p* < 0.01). Among KT patients, the seroconversion rate increased significantly from 10% after V2 to 39% after V3 (*p* < 0.01).

**Table 2:** SARS-CoV-2-specific humoral and cellular immune responses in KT, PD, and HD patients and healthy controls vaccinated with 2 doses of inactivated SARS-CoV-2 vaccine (V2) followed by a third dose of ChAdOx1 nCoV-19 vaccine (V3).

Immunogenicity N (%) or median (IQR)	Controls (N = 16)		HD (N = 31)		PD (N = 28)		KT (N = 31)	
	V2	V3	V2	V3	V2	V3	V2	V3
Anti-RBD IgG level (BAU/mL)	250.9 (90.9–612.2)	2209.7 (1494.3–2806.1)*	85.3 (33–412.1)	1740.9 (1106–3762.3)*	80.9 (13.3–146.7)	1092.5 (606.9–1927.2)*	0.3 (0.2–0.5)	1.0 (0.4–26.8)*
Anti-RBD IgG level ≥ 7.1 BAU/mL (Seroconversion)	16 (100)	16 (100)	29 (94)	31 (100)	23 (82)	28 (100)	3 (10)	12 (39)*^,a^^,^**
% neutralization by sVNT	74.6 (54.5–94.4)	99.5 (99.1–99.6)*	47.9 (13.5–85.4)	99.4 (98.8–99.7)*	40.1 (12.6–70.5)	98.8 (95.9–99.5)*	0 (0–0)	0.9 (0–9.9)*
Positive sVNT (threshold ≥ 35%)	15 (94)	16 (100)	19 (62)	31 (100)^a^^,^*	15 (54)	28 (100)^a^^,^*	0 (0)	5 (16)^a^^,^**
S1-specific T-cells, SFUs/10^6^ PBMCs	36 (18–79)	59 (27–167)	48 (12–100)	188 (32–480)	102 (25–222)	242 (71–473)	24 (0–80)	12 (0–60)
S1-specific T cell responders (threshold ≥ 6 SFUs/10^6^ PBMCs)	14 (88)	16 (100)	24 (77)	28 (90)	26 (93)	27 (96)	21 (68)	18 (58)^a^^,^**
SNMO-specific T-cells SFUs/10^6^ PBMCs	38 (23–112)	70 (16–184)	92 (12–176)	144 (24–300)	168 (28–325)	240 (56–467)	27 (4–116)	26 (4–67)
SNMO-specific T cell responders (threshold ≥ 6 SFUs/10^6^ PBMCs)	14 (88)	16 (100)	24 (77)	28 (90)	24 (86)	27 (96)	22 (71)	23 (74)^a^^,^**

*p < 0.05 (compared within group).

**p < 0.03 (compared with controls).

^a^Fisher’s exact test.

*BAU* binding antibody units, *HD* hemodialyzed patients, *IgG* immunoglobulin G, *IQR* interquartile range, *KT* kidney transplant recipients, *N* number, *PBMCs* peripheral blood mononuclear cells, *PD* peritoneal dialyzed patients, *RBD* receptor-binding domain, *S1* S1 domain of the spike protein, *SFU* spot forming unit, *SNMO* peptide pool of spike protein, nucleoprotein, membrane protein and open reading frame proteins, *sVNT* surrogate virus neutralization test.

The median % inhibition by neutralizing antibodies among the KT, PD, HD, and control, as measured by the neutralization (NT) test was greater at two weeks after V3 than two weeks after V2 in all groups (*p* < 0.05) (Table 2, Fig. 1c). The % NT in the PD, HD, and control groups was comparable but significantly lower in KT patients (*p* < 0.05). The rate of NT positivity was 100% in PD, HD, and control groups and 16% in the KT group. After V3, the seropositivity rate for neutralizing antibodies increased in ESKD patients (58% for V2 vs. 100% for V3, *p* < 0.01); all twenty-four ESKD patients with % NT below 35 after V2 became seropositive after V3. Similarly, KT recipients achieved a higher seropositive rate after V3 (16%) compared with 0% after V2 (*p* = 0.05).

The seroconversion rate for anti-RBD antibodies was higher in KT recipients who were maintained on a low therapeutic dose of MPA or MPA-sparing regimen compared with those who were maintained on a high mycophenolic acid dose (75% vs. 16%; odds ratio [OR], 10.7; 95% confidence interval [CI], 1.9–59.6; *p* < 0.01) **(**Supplementary Table 1). This association was significant for KT recipients who were NT positive (100% vs. 31%; OR, 23.9; 95% CI, 1.2–484.2; *p* < 0.05). Time after transplant, age < 50 years, lymphopenia (< 1.5 × 10^3^/μL), and low C_0_ level for CNI or CNI-sparing regimen were not significantly different between KT recipients with and without seroconversion and NT positivity.

### SARS-CoV-2 specific CMI responses

The median SFUs for S1-specific T-cell responses were significantly increased after V3 in PD and HD patients (242 [71–473] and 188 [32–480] T-cells/10^6^ PBMCs, respectively; *p* < 0.05) compared with those after V2. However, SFUs after V2 and V3 were not significantly different in the KT and control groups (*p* > 0.05) (Fig. 1d). The rates of S1-specific T-cell responders were 58%, 96%, 90%, 100% in KT, PD, HD, and controls, respectively.

After V3, the median SFUs for SNMO-specific T cell responses were significantly increased in HD patients compared with those after V2 (*p* < 0.05) but were not significantly different in the KT, PD, and control groups (12 [0–60], 240 [56–467], and 70 [16–184] T-cells/10^6^ PBMCs, *p* > 0.05). The rates of SNMO-specific T cell responders were 74%, 96%, 90%, and 100% in the KT, PD, HD, and control groups, respectively, after V3. The responder rate in the control group was higher than that in the KT group (*p* < 0.05).

No significant differences were observed for low therapeutic doses of mycophenolic acid or mycophenolic acid-sparing regimen, time after transplant, age < 50 years, lymphopenia (< 1.5 × 10^3^/μL), low C_0_ level of calcineurin inhibitors, or CNI-sparing regimen between KT recipients with and without S1-specific T-cell responses.

### Adverse events

**Figure 2:**
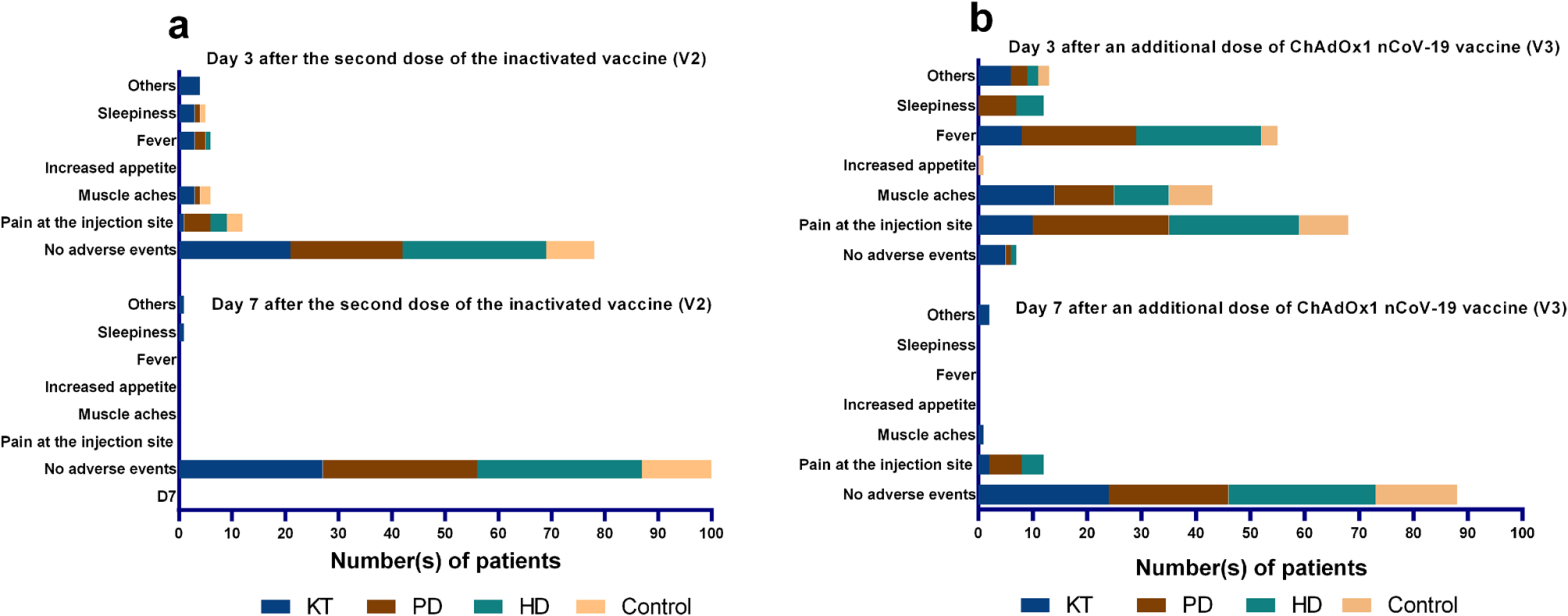
Solicited adverse events on days 3 and 7 after (**a**) the second dose of inactivated SARS-CoV-2 vaccine (V2) and (**b**) the additional dose of ChAdOx1 nCoV-19 vaccine (V3). HD, hemodialysis; KT, kidney transplant; PD, peritoneal dialysis; SARS-CoV-2, severe acute respiratory syndrome coronavirus-2.

There were no serious local or systemic AEs within 30 min after V3. Solicited AEs within 3 and 7 days after V3 are presented and compared with those after V2^7,8^ in Fig. 2 and Supplementary Table 2. AEs were reported in the KT (82%), PD (96%), HD (97%), and control (100%) groups within 3 days after V3. The numbers of AEs were significantly greater after V3 compared with those after V2 across all groups (*p* < 0.05). The most common AEs included pain at the injection site (9–39%), muscle aches (9–32%), fever (3–19%), and sleepiness (0–6%). On day 7, the AEs were almost entirely resolved. All AEs were graded as mild. No acute rejection episodes occurred in vaccinated KT recipients. No unsolicited AEs were reported.

## Discussion

This study evaluated immune responses in dialysis and KT patients following a third dose of ChAdOx1 nCoV-19 vaccine, administered 4–8-weeks after two doses of an inactivated SARS-CoV-2 vaccine. This regimen induced robust SARS-CoV-2–specific HMI responses, as determined by measuring the levels of anti-RBD IgG and % NT, and achieved the same rate of seroconversion and NT positivity among ESKD patients as immunocompetent individuals. Although the magnitude of HMI and response rates were improved with this heterologous vaccination strategy, HMI remained suboptimal in KT recipients. A similar result was observed for SARS-CoV-2-specific CMI, which was assessed by IFN-γ ELISpot responses against SARS-CoV-2 S1 protein. Furthermore, most patients only experienced mild and spontaneously resolved AEs with this new vaccine strategy.

Few studies have reported HMI responses after three doses of COVID-19 vaccine among ESKD patients, and these studies focused on the same vaccine platform^19–22^. We demonstrated that the addition of ChAdOx1 nCoV-19 vaccines enabled ESKD patients to produce remarkably higher antibody levels which could potentially act against the variants. This heterologous inactivated virus/ChAdOx1 nCoV-19 vaccination regimen generated an excellent level of seroconversion in non-responders after V2, which was comparable to the efficacy of three doses of the BNT162b2 mRNA vaccine^19–22^. The magnitude of anti-RBD antibody production and the % NT in ESKD patients were comparable to the control group. However, there was a lower trend of production in the PD subgroup. This relationship was observed for NT positivity and T-cell immune responses. A significant proportion of T-cell responders (> 90%) were observed in the PD, HD, and control groups. The inactivated virus/ChAdOx1 nCoV-19 vaccination regimen enhanced the % NT in all dialysis patients who had previously produced a % NT below the cut-off level, and these patients became seropositive after the additional dose. Furthermore, the addition of the ChAdOx1 nCoV-19 vaccine after inactivated vaccine improved the % NT in ESKD patients to a level comparable to that of healthy controls. This effect concurs with the efficacy of two doses of BNT162b2 reported by Carr et al.^23^. Although direct comparisons between studies are limited due to different assays, timepoints and patient populations, the rate of NT seropositivity with the inactivated virus/ChAdOx1 nCoV-19 regimen was equal to or higher than that observed for two doses of the BNT162b2 vaccine^23,24^. Feng et al. reported that the anti-RBD antibody levels above 506 BAU/mL correlated with 80% vaccine efficacy against symptomatic COVID-19 infection from the Alpha (B.1.1.7) variant. Although we could potentially use this cut-off value to extrapolate the efficacy of new vaccine regimens, variability of ongoing spreading variant and individual immune status could limit interpretation. Nevertheless, most participants could achieve anti-RBD antibody levels more remarkable than this number, excluding KT recipients^25^. Thus, ESKD patients who received a two-dose regimen of inactivated vaccine may benefit from this heterologous combination.

Among several COVID-19 vaccines available, a third dose of an mRNA-based vaccine appeared to substantially improve the prevalence of anti–SARS-CoV-2 antibodies from 40 to 68% after a regular 2-dose course in solid organ transplant (SOT) recipients^26–29^. KT recipients who received an adenovirus-vectored vaccine revealed a significantly impaired immune response rate of 44% after a full course^30^. Data regarding an additional dose after an entire course of the adenovirus-vectored vaccine in SOT recipients remains limited. Among KT recipients in this study, the seroconversion rate after the heterologous vaccine regimen was 39% compared with 49% and 26% in KT recipients fully vaccinated with mRNA-1273 and BNT162b2, respectively^30^. Although no threshold has been established for protective immunity, antibody levels and their neutralization properties were lower in KT transplant recipients than in the control group who received the same vaccine regimen. Two doses of inactivated vaccine resulted in low immune responses among KT recipients as has been previously reported^7,31^. A supplemental dose of the adenovirus-vectored vaccine in these immunosuppressed patients successfully increased the seroconversion rate from 10% to approximately 40% and NT positivity in up to one out of six patients, which has not been reported. Therefore, our study supported the WHO interim recommendation for an additional vaccine in patients who are immunocompromised^9^. We propose that the accumulation of data after switching between vaccine platforms may be valuable for designing optimal vaccine regimens for patients with comorbidities and those receiving immunosuppressants.

Since measurement of cell-mediated immune response may not be widely available due to lacking the commercialized assay, we explored whether the association between vaccine-elicited humoral and cellular immune response exists in all participants, using Spearman’s correlation analysis. There was a weak positive correlation (*r* = 0.23, *p* = 0.02) between the anti-RBD antibody levels and the S1-specific T-cell responses after the second dose of inactivated SARS-CoV-2 vaccine and a moderate positive correlation (*r* = 0.39, *p* < 0.001) between those two parameters after the additional dose of ChAdOx1 nCoV-19 vaccine (Supplementary Fig. 7).

Several factors may lead to a failure in seroconversion after vaccination in transplant recipients, including higher numbers of immunosuppressive drugs^29^ and a dose of mycophenolate mofetil > 1 g/day^7,32^. In our study, KT patients with HMI responses received ≤ 1 g/day MMF, ≤ 720 mg/day MPS, or were on a treatment regimen without MPA, which reaffirmed the impact of MPA on seroconversion observed in a previous study^6^. Although immunosuppressive agents may have significantly blunted HMI in our KT patients, this effect was not demonstrated for CMI. A similar phenomenon was observed after a single dose of BNT162b2 in KT recipients^11^. A recommendation for withholding MPA after COVID vaccination exists for patients with autoimmune diseases^33^, and a strategy regarding MPA dose reduction among SOT recipients is undergoing investigation^34^.

The present study evaluated AEs after this new heterologous vaccine regimen in immunocompromised individuals. This specific vaccination strategy appeared to be tolerable and relatively safe. Although a substantial proportion of our participants experienced at least one AE after an additional dose of vaccine, the AEs were graded as non-severe. Pain at the injection site was frequently reported after the ChAdOx1 nCoV-19 vaccination in a phase 3 trial^35^.

Our study had limitations, including a relatively small number of patients and unmatched controls^36^. The sVNT is a surrogate test for neutralizing antibodies, which is not as standardized as the plaque reduction neutralization test, and the assumption of protective immunity against infection from this vaccine regimen cannot be concluded. Moreover, immune escape from vaccine-elicited immunity by the variant of concerns in circulation has been reported^37^. Studies using the live virus-based neutralization assay against variants of concerns are required to inform vaccine efficacy^36^. Additionally, the effectiveness may be variable based on local virus transmission rates and different COVID-19 variants. Furthermore, a direct comparison of immunogenicity may not be possible because of different vaccine platforms, variable definitions of immune responses, and intra- and inter-individual differences. Therefore, clinicians should interpret and apply these pieces of information with caution. Lastly, since we only screened SARS-CoV-2 infection by medical history, there is a possibility that our participants had been infected before receiving a third ChAdOx1 nCoV-19 dose. However, our findings provide additional information on SARS-CoV-2–specific immune responses following this unique regimen and the potential utilization of this strategy when mRNA-based vaccines are inaccessible. Our study assessed CMI, which is believed to boost a prolonged protective memory response, especially T-cell-dependent B-cell activation, which should be further explored^24^. Furthermore, although information regarding short-term AEs was collected, long-term safety and potential indirect AEs warrant further studies.

With acceptable short-term AEs, the heterologous 3-dose SARS-CoV-2 vaccination regimen elicited greater immunogenicity among dialyzed patients; however, inadequate responses remained in the KT recipients. Therefore, we conclude that an additional vaccine dose should be considered to a primary series to produce greater immune responsiveness among ESKD and KT patients, who remain vulnerable to COVID-19-related disease and mortality.

## Supplementary Information

Supplementary Information.

## Data Availability

The datasets generated during and/or analyzed during the current study are not publicly available because of privacy and ethical restrictions, but anonymized data are available from the corresponding author on reasonable request.
