# Supplementary material for: Immunogenicity of ChAdOx1 nCoV-19 vaccine after a two-dose inactivated SARS-CoV-2 vaccination of dialysis patients and kidney transplant recipients
Source: Sci Rep. 2022 Mar 4;12:3587. doi: 10.1038/s41598-022-07574-w (PMC8897448; doi:10.1038/s41598-022-07574-w)
Supplement: Supplementary file 1 — Supplementary Information. [file 41598_2022_7574_MOESM1_ESM.pdf]

Supplementary figures and tables

Immunogenicity of ChAdOx1 nCoV-19 Vaccine after a Two-dose Inactivated SARS-CoV-2 Vaccination of Dialysis Patients and Kidney Transplant Recipients

Jackrapong Bruminhent[1,2], Chavachol Setthaudom[3], Rungthiwa Kitpermkiat[4], Sasisopin Kiertiburanakul[1], Kumthorn Malathum[1], Montira Assanatham[2,4], Arkom Nongnuch[2,4], Angsana Phuphuakrat[1], Pongsathon Chaumdee[5], Chitimaporn Janphram[6], Sansanee Thotsiri[6], Piyatida Chuengsaman[7], Sarinya Boongird*[2,4]

[1]Division of Infectious Diseases, Department of Medicine, Faculty of Medicine Ramathibodi Hospital, Mahidol University, Bangkok, Thailand

[2]Ramathibodi Excellence Center for Organ Transplantation, Faculty of Medicine Ramathibodi Hospital, Mahidol University, Bangkok, Thailand

[3]Immunology Laboratory, Department of Pathology, Faculty of Medicine Ramathibodi Hospital, Mahidol University, Bangkok, Thailand

[4]Division of Nephrology, Department of Medicine, Faculty of Medicine Ramathibodi Hospital, Mahidol University, Bangkok, Thailand

[5]Department of Medicine, Faculty of Medicine Ramathibodi Hospital, Mahidol University, Bangkok, Thailand

[6]Somdech Phra Debaratana Medical Center, Faculty of Medicine Ramathibodi Hospital, Mahidol University, Bangkok, Thailand

[7]Banphaeo-Charoenkrung Peritoneal Dialysis Center, Banphaeo Dialysis Group, Banphaeo Hospital, Bangkok, Thailand

***Corresponding author**

Sarinya Boongird, MD

Division of Nephrology, Department of Medicine, and the Ramathibodi Excellence Center for Organ Transplantation, Faculty of Medicine Ramathibodi Hospital, Mahidol University, 270 Rama VI Road, Ratchathewi, Bangkok, 10400, Thailand

Email: sarinya.bon@mahidol.ac.th

Supplementary Figure 1. Study design

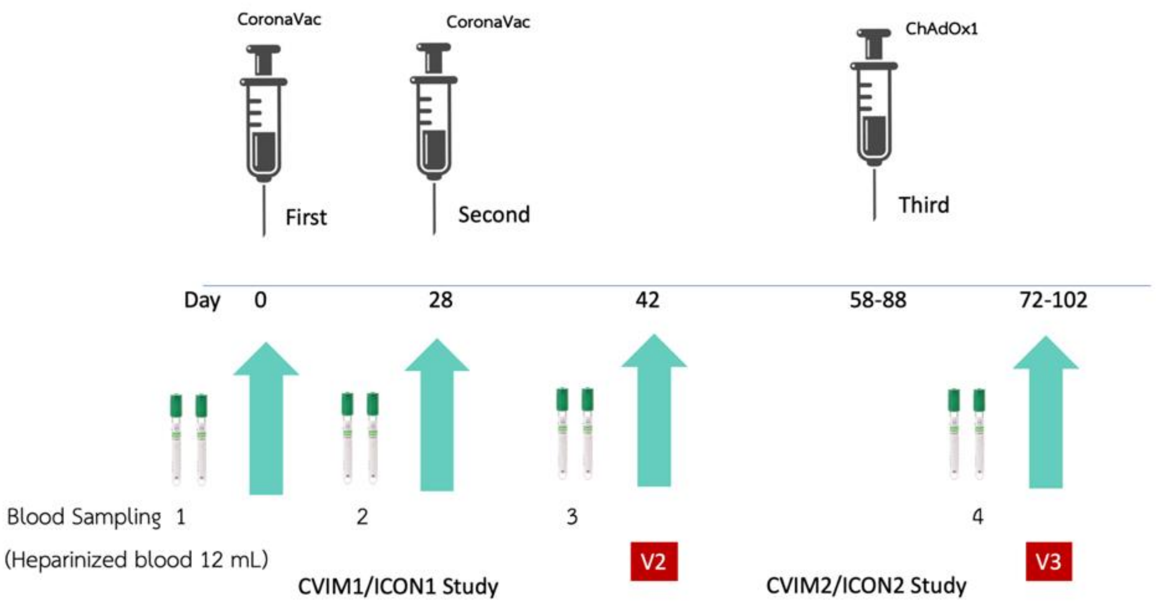

Supplementary Figure 2. Assessment sheet for immediate adverse events after vaccination

CVIM Project: Subject No.______________________ Version 1.0 170321

**Assessment of AE after vaccination 30 min. (Immediate AEs)**

Did any Solicited adverse event occurs within 30 minutes after vaccination?

1) Solicited local injection site reactions:

Pain	☐ None ☐ Grade1 (mild) ☐ Grade2 (severe without action) ☐Grade3 (severe with action)
Redness	☐ None ☐ Yes, ______mm
Swelling.	☐ None ☐ Yes, ______mm
**Action Taken:**	☐ None ☐ Medication ☐ Health Care Provider Contact ☐ Hospitalized ☐ Discontinuation of Study vaccination

2) Solicited systemic adverse events

Headache	☐ None ☐ Grade1 (mild) ☐ Grade2 (severe without action) ☐Grade3 (severe with action)
Malaise	☐ None ☐ Grade1 (mild) ☐ Grade2 (severe without action) ☐Grade3 (severe with action)
Myalgia	☐ None ☐ Grade1 (mild) ☐ Grade2 (severe without action) ☐Grade3 (severe with action)
**Action Taken:**	☐ None ☐ Medication ☐ Health Care Provider Contact ☐ Hospitalized ☐ Discontinuation of Study vaccination

Note:____

____

Investigator's initial: ____________________________**Date:** ____/_____/______

Supplementary Figure 3. Assessment sheet for solicited adverse events after vaccination

CVIM Project: Subject No.______________________ Version 1.0 170321

Phone call for solicited adverse events

First name _______________ Last name________________

First dose date____________________

Second dose date____________________

	Dose	Date	Time	Symptom		Date of relief	Medication and dosing	Date of treatment	Date of discontinuation	Investigator's initial
Day 3	1			-pain at injection site<br>☐ mild<br>☐ severe without medication<br>☐ severe with medication<br>-redness at injection site<br>☐ yes size_____mm.<br>-swelling at injection site<br>☐yes size_____mm.	-headache<br>☐ mild<br>☐ severe without medication<br>☐ severe with medication<br>-malaise<br>☐ mild<br>☐ severe without medication<br>☐ severe with medication<br>-myalgia<br>☐ mild<br>☐ severe without medication<br>☐ severe with medication					
Day 7	1			-pain at injection site<br>☐ mild<br>☐ severe without medication<br>☐ severe with medication<br>-redness at injection site<br>☐ yes size_____mm.<br>-swelling at injection site<br>☐yes size_____mm.	-headache<br>☐ mild<br>☐ severe without medication<br>☐ severe with medication<br>-malaise<br>☐ mild<br>☐ severe without medication<br>☐ severe with medication<br>-myalgia<br>☐ mild					

Supplementary Figure 4. Assessment sheet for unsolicited adverse events after vaccination

CVIM Project: Subject No.______________________ Version 1.0 170321

Diary for unsolicited adverse events

Please call +66-9-9154-9661 for any concerns you may have

First name ________________ Last name_________________

First dose date_____________________

Second dose date_____________________

Date	Time	Symptoms	Date of onset	Date of relief	Medication and dosing	Start date	Stop date	Comments

Supplementary Figure 5. Inclusion and exclusion criteria

Inclusion criteria

1. Aged 18-59 years old
2. Received two doses of inactivated SARS-CoV-2 vaccine
3. Previously included in CVIM 1 study (Bruminhent J, et al. doi: 10.1111/ajt.16867.) and ICON 1 study (Boongird S, et al. doi: 10.1007/s40121-021-00574-9.)
4. Comply with blood collection 4-6 weeks after vaccination
5. Can provide informed consent
6. At least one-month post-transplant (kidney transplant patients)
7. Stable allograft function and immunosuppressive regimen (kidney transplant patients)
8. Stable dialysis prescriptions for at least one month (Dialysis patients)

Exclusion criteria

1. Patients with suspected respiratory tract infection in the preceding 3 days
2. Concurrent active infection
3. Recent diagnosis of allograft rejection requiring intense immunosuppressants (kidney transplant patients) (intense immunosuppressants included methylprednisolone pulse therapy with 500 mg IV daily for 3 days, antithymocyte globulin therapy within 3 months, rituximab therapy within 6 months, or prednisolone more than 15 mg/day)
4. Receiving other vaccination within 4 weeks
5. Previous history of COVID-19
6. Prior administration of COVID-19 vaccine

Supplementary Figure 6. Consort diagram including number of participants who were included in the study

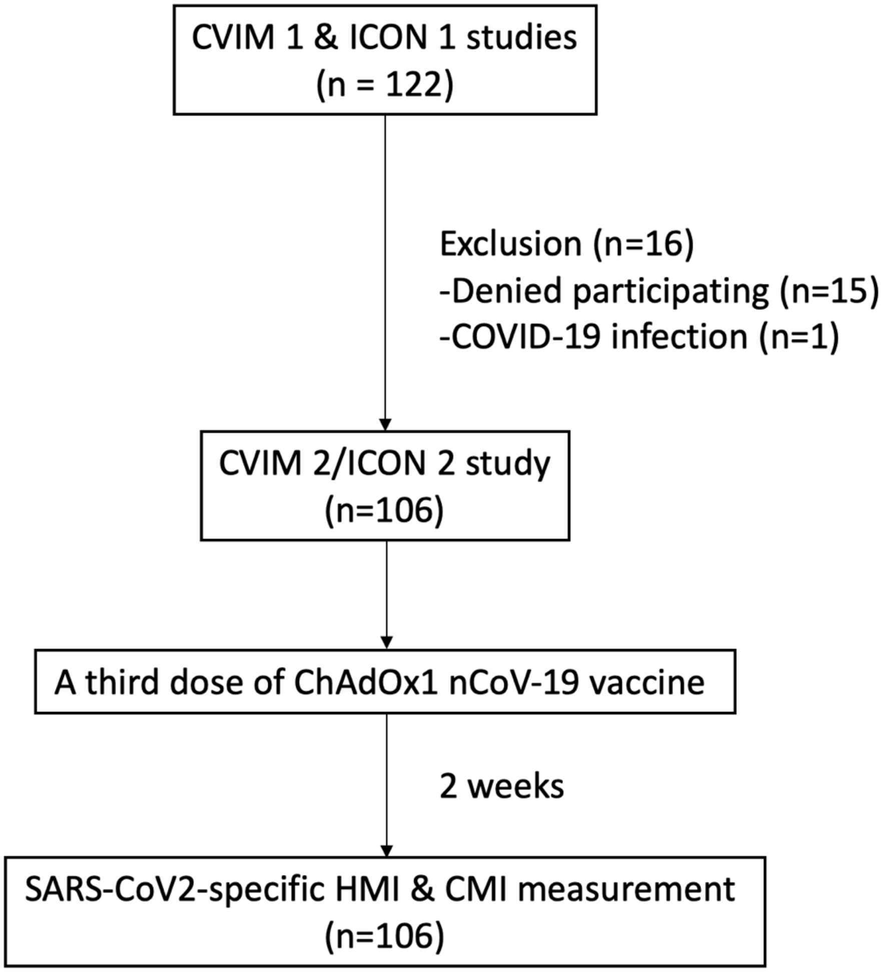

Supplementary Figure 7. Correlation between anti-receptor binding domain immunoglobulin G levels and S1-specific T-cell counts measured at 2 weeks after (a) the second dose of inactivated SARS-CoV-2 vaccine (V2) and (b) the additional dose of ChAdOx1 nCoV-19 vaccine (V3). Each dot represents an individual participant. Correlation strength and significance were calculated using Spearman's correlation coefficient.

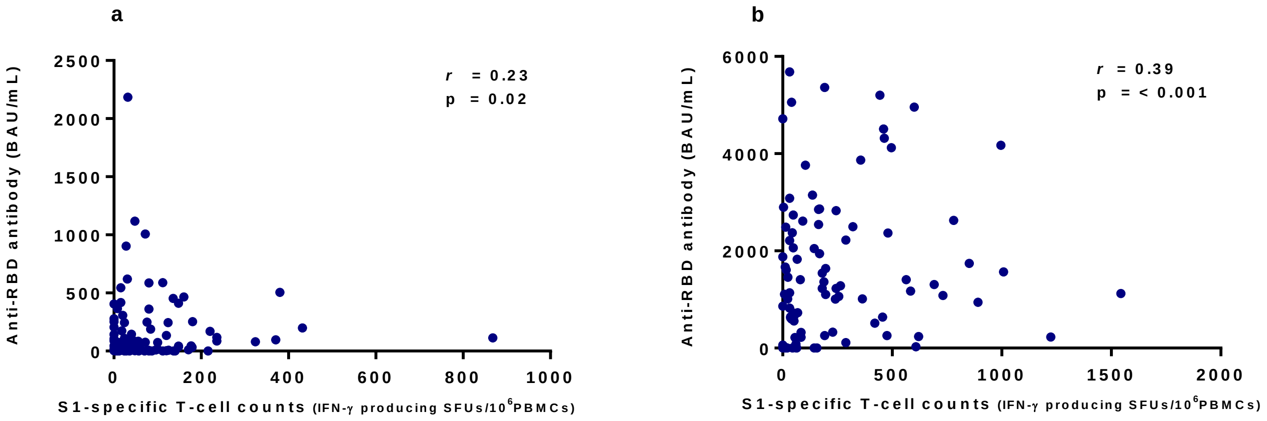

Supplementary Table 1. Characteristics of kidney transplant recipients with and without seroconversion after the third dose of ChAdOx1 nCoV-19 vaccine

Factors<br>N (%)	Seroconversion<br>N = 12	No seroconversion<br>N = 19	p value
Age, < 50 years	4 (33)	10 (53)	0.29
Male	8 (67)	10 (53)	0.44
Time after transplant (< 1 year)	0 (0)	2 (5)	0.51[a]
Tacrolimus-based regimen	6 (50)	13 (68)	0.31
Cyclosporine-based regimen	5 (42)	5 (26)	0.49
Low C_0 level of calcineurin inhibitors (tacrolimus $\leq$ 5 ng/mL or cyclosporine $\leq$ 150 ng/mL) or CNI-sparing regimen	7 (58)	7 (37)	0.28
Mycophenolic acid-based regimen	11 (97)	18 (95)	0.73
Low therapeutic dose of mycophenolic acid (mycophenolate mofetil $\leq$ 1 g/day or mycophenolate sodium $\leq$ 720 mg/day) or mycophenolic acid-sparing regimen	8 (75)	3 (16)	0.03
Absolute lymphocyte count < 1.5 × 10^3/μL	3 (25)	3 (16)	0.65
Albumin < 4 g/dL	2 (17)	5 (26)	0.53

BAU, binding antibody unit

[a]Fisher's exact test

Supplementary Table 2. Solicited adverse events on days 3 and 7 after vaccination with two doses of inactivated SARS-CoV-2 vaccine (V2) and a third dose of the ChAdOx1 nCoV-19 vaccine (V3).

Group N (%)	Days post-V2		Days post-V3	
	Day 3	Day 7	Day 3	Day 7
**Kidney transplant recipients**[a]				
Adverse events	7 (25)	1 (4)	23 (82)*	4 (14)
Pain at the injection site	1 (3)	0 (0)	10 (23)	2 (7)
Muscle aches	3 (9)	0 (0)	14 (32)	1 (3)
Increased appetite	0 (0)	0 (0)	0 (0)	0 (0)
Fever	3 (9)	0 (0)	8 (19)	0 (0)
Sleepiness	3 (9)	1 (3)	0 (0)	0 (0)
Other	4 (12)	1 (3)	6 (14)	2 (7)
**Peritoneal dialyzed patients**				
Adverse events	8 (28)	0 (0)	27 (96)	6 (21)
Pain at the injection site	5 (17)	0 (0)	25 (89)	6 (21)
Muscle aches	1 (3)	0 (0)	11 (39)	0 (0)
Increased appetite	0 (0)	0 (0)	0 (0)	0 (0)
Fever	2 (6)	0 (0)	21 (75)	0 (0)
Sleepiness	1 (3)	0 (0)	7 (25)	0 (0)
Other	0 (0)	0 (0)	3 (11)	0 (0)
**Hemodialyzed patients**				
Adverse events	4 (13)	0 (0)	30 (97)	4 (13)

Pain at the injection site	3 (10)	0 (0)	24 (77)	4 (13)
Muscle aches	0 (0)	0 (0)	10 (32)	0 (0)
Increased appetite	0 (0)	0 (0)	0 (0)	0 (0)
Fever	1 (3)	0 (0)	23 (74)	0 (0)
Sleepiness	0 (0)	0 (0)	5 (16)	0 (0)
Other	0 (0)	0 (0)	2 (6)	0 (0)
**Controls**[C]				
Adverse events	6 (40)	0 (0)	15 (100)[*,b]	0 (0)
Pain at the injection site	3 (20)	0 (0)	9 (60)	0 (0)
Muscle aches	2 (13)	0 (0)	8 (53)	0 (0)
Increased appetite	0 (0)	0 (0)	1 (6)	0 (0)
Fever	0 (0)	0 (0)	3 (20)	0 (0)
Sleepiness	1 (7)	0 (0)	0 (0)	0 (0)
Other	0 (0)	0 (0)	2 (13)	0 (0)

*$p < 0.05$

[a]Evaluated in 28 participants

[b]Fisher's exact test

[c]Evaluated in 15 participants
